# RETRACTION: MicroRNA‐16 inhibits glioma cell growth and invasion through suppression of BCL2 and the nuclear factor‐κB1/MMP9 signaling pathway

**DOI:** 10.1111/cas.16269

**Published:** 2024-07-22

## Abstract

**Retraction**: T.‐Q. Yang, X.‐J. Lu, T.‐F. Wu, D.‐D. Ding, Z.‐H. Zhao, G.‐L. Chen, X.‐S. Xie, B. Li, Y.‐X. Wei, L.‐C. Guo, Y. Zhang, Y.‐L. Huang, Y.‐X. Zhou, and Z.‐W. Du, “MicroRNA‐16 inhibits glioma cell growth and invasion through suppression of BCL2 and the nuclear factor‐κB1/MMP9 signaling pathway,” *Cancer Science* 105 no. 3 (2014): 265–271. https://doi.org/10.1111/cas.12351.

The above article, published online on 13 January 2014 in Wiley Online Library (wileyonlinelibrary.com), has been retracted by agreement between the journal Editor‐in‐Chief, Masanori Hatakeyama; the Japanese Cancer Association; and John Wiley and Sons Ltd. The journal had previously published a correction regarding duplicated portions of Figure 4a on 24 May 2022. Following publication of this correction, the journal was made aware that not all duplicated portions of Figure 4a had been replaced. Additionally, concerns were raised of splicing in Figure 2d and image duplications in Figures 2d, 3d, and 4c. The authors did not respond to inquiries from the publisher regarding these concerns. and did not provide the requested original images and data. Therefore, the data reported in this article are considered unreliable and the journal must issue a retraction. The authors did not respond to our notice of retraction.

**Retraction**: T.‐Q. Yang, X.‐J. Lu, T.‐F. Wu, D.‐D. Ding, Z.‐H. Zhao, G.‐L. Chen, X.‐S. Xie, B. Li, Y.‐X. Wei, L.‐C. Guo, Y. Zhang, Y.‐L. Huang, Y.‐X. Zhou, and Z.‐W. Du, “MicroRNA‐16 inhibits glioma cell growth and invasion through suppression of BCL2 and the nuclear factor‐κB1/MMP9 signaling pathway,” *Cancer Science* 105 no. 3 (2014): 265–271. https://doi.org/10.1111/cas.12351.

The above article, published online on 13 January 2014 in Wiley Online Library (wileyonlinelibrary.com), has been retracted by agreement between the journal Editor‐in‐Chief, Masanori Hatakeyama; the Japanese Cancer Association; and John Wiley and Sons Ltd. The journal had previously published a correction regarding duplicated portions of Figure 4a on 24 May 2022. Following publication of this correction, the journal was made aware that not all duplicated portions of Figure 4a had been replaced. Additionally, concerns were raised of splicing in Figure 2d and image duplications in Figures 2d, 3d, and 4c. The authors did not respond to inquiries from the publisher regarding these concerns. and did not provide the requested original images and data. Therefore, the data reported in this article are considered unreliable and the journal must issue a retraction. The authors did not respond to our notice of retraction.

